# OLFM4, KNG1 and Sec24C identified by proteomics and immunohistochemistry as potential markers of early colorectal cancer stages

**DOI:** 10.1186/s12014-017-9143-3

**Published:** 2017-03-24

**Authors:** Florence Quesada-Calvo, Charlotte Massot, Virginie Bertrand, Rémi Longuespée, Noëlla Blétard, Joan Somja, Gabriel Mazzucchelli, Nicolas Smargiasso, Dominique Baiwir, Marie-Claire De Pauw-Gillet, Philippe Delvenne, Michel Malaise, Carla Coimbra Marques, Marc Polus, Edwin De Pauw, Marie-Alice Meuwis, Edouard Louis

**Affiliations:** 1 Gastroenterology Department, GIGA-R, Liège University Hospital CHU, ULg, GIGA CHU-B34 Avenue de l’Hôpital 11, 4000 Liège, Belgium; 2 Laboratory of Mass Spectrometry, Chemistry Department, GIGA-R, CART, ULg, 4000 Liège, Belgium; 3 Department of Anatomy and Pathology, GIGA-R, Liège University Hospital CHU, ULg, 4000 Liège, Belgium; 4 GIGA Proteomic Facility, ULg, 4000 Liège, Belgium; 5 Mammalian Cell Culture Laboratory, Department of Preclinical and Biomedical Sciences, GIGA-R, ULg, 4000 Liège, Belgium; 6 Department of Clinical Sciences, Rheumatology, Liège University Hospital CHU, 4000 Liège, Belgium; 7 Abdominal Surgery Department, University Hospital CHU, 4000 Liège, Belgium

## Abstract

**Background:**

Despite recent advances in colorectal cancer (CRC) diagnosis and population screening programs, the identification of patients with preneoplastic lesions or with early CRC stages remains challenging and is important for reducing CRC incidence and increasing patient’s survival.

**Methods:**

We analysed 76 colorectal tissue samples originated from early CRC stages, normal or inflamed mucosa by *label*-*free proteomics*. The characterisation of three selected biomarker candidates was performed by immunohistochemistry on an independent set of precancerous and cancerous lesions harbouring increasing CRC stages.

**Results:**

Out of 5258 proteins identified, we obtained 561 proteins with a significant differential distribution among groups of patients and controls. KNG1, OLFM4 and Sec24C distributions were validated in tissues and showed different expression levels especially in the two early CRC stages compared to normal and preneoplastic tissues.

**Conclusion:**

We highlighted three proteins that require further investigations to better characterise their role in early CRC carcinogenesis and their potential as early CRC markers.

**Electronic supplementary material:**

The online version of this article (doi:10.1186/s12014-017-9143-3) contains supplementary material, which is available to authorized users.

## Background

Colorectal cancer (CRC) is the third leading cause of death by cancer in the western world. Population screening programs for identification of patients with preneoplastic lesions or early CRC stages is important for reducing CRC incidence and for increasing patient’s survival. CRC shows a multi-stage progression with sequential accumulation of genetic alterations that might occur over a long period of time and with absence of symptoms [1]. Colonoscopy is the method of choice for the diagnosis of CRC [2]. It can also be used for broad population screening but is hampered in this setting by its cost, discomfort and risks for the patients. Different non-invasive tests as faecal occult blood test (FOBT) and faecal immunochemical test (FIT) are used for CRC screening [3], but their use is not generalised in all countries. Indeed, despite that some of these are cost-effective, they often lack of sensitivity as well as specificity. Hence early non invasive specific and sensitive markers are still awaited for screening purposes.

Recent advances in genomics, proteomics and metabolomics have increased the list of potential biomarkers associated with CRC and contributed to our understanding of its development [4]. Due to the heterogeneous nature of CRC, a biomarker signature (panel of several proteins) may be more effective for screening test development than a single biomarker.

Dysplastic and neoplastic tissues regulate protein expression and produce protein profiles that might be correlated to precancerous or cancerous specific progression. To capture these events, it is adequate to investigate protein changes directly in the colon mucosa. Formalin-fixed paraffin-embedded (FFPE) tissues stored in hospital biobanks represent a valuable resource for retrospective analysis as larger populations can be studied, enhancing the probability to identify significant and specific potential CRC biomarkers.

Hence our aim was to screen FFPE specimens including early CRC stages using label free proteomics. We validated three selected proteins by immunohistochemistry (IHC) on a larger independent sample set containing normal tissue, CRC and precancerous lesions for their full histological characterisation and for partial validation of the proteomic results obtained.

## Methods

### Patients and FFPE tissue samples

Human FFPE tissue blocks were obtained from the Biobank of the Liège University Hospital, Belgium. This study received approval by the Ethics reviewing board of the University Hospital of Liège, Belgium (15 October 2014) (internal ref number: 2005-144). The clinical and pathological characteristics of the patients are summarized in Table 1 for the patients included in the proteomic analysis (left panel) and for the patients used for the IHC validation (right panel). We selected for the differential proteomic analyses some tissue samples from early CRC stages (namely pT1N0M0 and pT2N0M0) and compared these to normal and inflamed tissues taken from diverticular diseases. We characterised IHC tissue distributions of some selected proteins in all possible precancerous and cancerous stages, from adenomas with low grade dysplasia to pT4NM of CRC. Different exclusion criteria were applied: no other digestive disease or cancer 6 months before or after resection, no hereditary nonpolyposis CRC and no familial adenomatous polyposis. The control group was composed of patients with diverticular diseases and the selected tissues treated were isolated within the diverticulitis zone itself [named diverticulitis inflammatory (DI)] or in the adjacent normal tissue [named diverticulitis healthy (DH)]. All pathological stages (normal, inflammatory, adenomas and cancers) were assessed according to clinical diagnosis standardised guidelines and according to the AJCC TNM staging system [5–8].

**Table 1:** Clinical information of patients included in the study

	Proteomics discovery set of patients			IHC characterisation set of patients							
	Diverticular disease	Adenocarcinoma		Diverticular disease	Adenoma		Adenocarcinoma				
	Healthy and paired inflammatory	pT1N0M0	pT2N0M0	Healthy and paired inflammatory	Low grade	High grade	pTis	pT1N0M0	pT2N0M0	pT3	pT4
Female n/male n (n total)	9/11 (20)	5/11 (16)	10/10 (20)	10/10 (20)	10/10 (20)	8/12 (20)	5/7 (12)	7/13 (20)	7/13 (20)	10/10 (20)	10/10 (20)
Age (years) median (range)	63 (51–73)	67 (52–87)	78 (61–87)	63 (41–84)	64 (55–72)	67 (48–92)	60 (45–87)	73 (54–91)	70 (55–93)	74 (54–42)	75 (39–89)
Localisation n											
Rectum	–	2	7	–	5	5	1	4	2	–	1
Sigmoid	18	2	2	18	4	5	8	8	10	5	4
Colon	2	8	5	2	8	8	2	5	6	11	13
Cecum	–	4	6	–	3	2	1	3	2	4	2
pN stage	–	–	–	NR	NR	NR	NR	NR	NR	9	11
pM stage	–	–	–	NR	NR	NR	NR	NR	NR	2	2
Percentage of tumoral cells mdian (range)	–	60% (40–80)	50% (10–80)	–	–	–	–	–	–	–	–

The proteomic discovery patient set (n = 56) is detailed in the left panel and the immunohistology set of patients (n = 152) is provided in the right panel. Each patient with a diverticular disease provided paired tissue samples located in two different regions: diverticular disease healthy, DH (n = 20) and inflammatory, DI (n = 20)

None of the CRC cases had been treated by chemotherapy or radiotherapy before surgical resection.

All the tissue specimens were processed using a standard procedure for formalin fixation (24 h) and were embedded in paraffin as done for routine clinical analysis [9]. Histological diagnosis of normal tissues, inflammation, adenocarcinoma and adenoma types and grades were confirmed by trained anatomopathologists (N.B., J.S.) after microscopic examination of the hematoxylin and eosin (H&E) stained sections.

### Pre-treatment of FFPE slices before the proteomic analysis

In order to obtain an enriched population of epithelial cells (either normal or neoplastic), the tissue sections (thickness 6 µm) were macrodissected. The percentage of tumoral cells was established by trained anatomopathologists (N.B., J.S.) and is communicated in Table 1. The H&E sections were scanned using Hamamatsu Photonics K.K. (magnification 40×) and uploaded into the CYTOMINE application [10] to measure the surface of the macrodissected Zones. A constant surface of tissue was processed per patient and correspond to 300 mm^2^.

### Proteomic discovery study

**Fig. 1:**
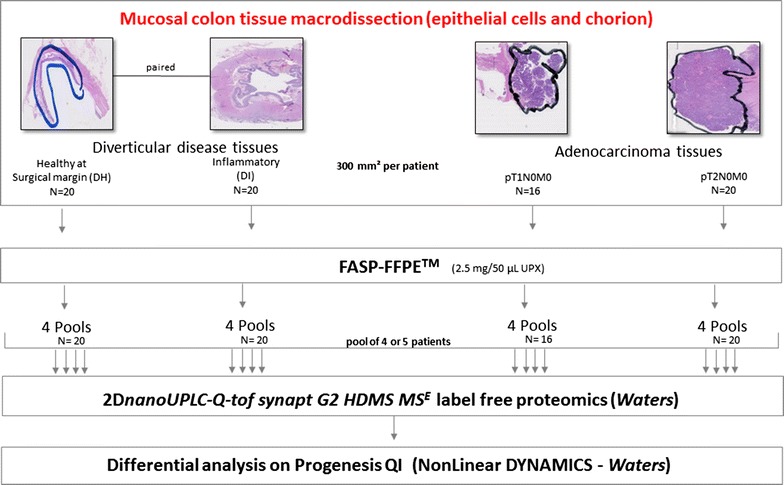
Proteomic discovery experiment workflow. Tissue section manual macrodissections (see line surrounding specific tissue area selected) were performed to obtain an enriched population of epithelial cells. The FASP-FFPE™ protein digestion kit was applied on each patient tissue (using around 300 mm^2^ of 6 µm tissue section). FASP-FFPE™ was applied on the dry material resuspended in the UPX buffer. Four to five patient sample digests were grouped per pool analysed. Four pools per disease groups were analysed by label-free proteomics differential analysis. *UPX* universal protein extraction

Figure 1 shows the workflow of the proteomic discovery experiment.

#### FFPE sample preparation

##### Slicing, deparaffinization and rehydration

FFPE sections for a total of 300 mm^2^ of macrodissected tissue per patient were treated as previously described [11]. Deparaffinization was performed by incubating the samples twice in xylene and followed by ethanol (100%) incubation (5 min each). Centrifugation was applied at 15,000 rpm for 5 min at room temperature between every 1 mL solvent changes. The material placed in the microtube was further evaporated to dryness with a speed-vacuum at room temperature for 5 min. The material was weighed before and after deparaffinization.

##### FFPE-FASP™ application

The tissue sections after deparaffinization were weighed dry and resuspended in the universal protein extraction buffer (UPX) provided in the FFPE-FASP*™* Protein Digestion Kit. All downstream steps of digestion and peptide extraction were performed using 2.5 mg of dry material resuspended in 50 mL of UPX buffer, according to the manufacturer instructions (Expedeon, Cambridge, UK). The protein concentration of each sample was determined using the RCDC Protein Assay Kit (BioRad, Hercules, CA, USA) before FFPE-FASP*™* treatment. For each disease category, four pools were composed of four–five patients using equal quantity of material of each individual sample protein digest (Fig. 1).

##### Sample conditioning and spiking with internal standards

The quantity of material present in each sample was determined using the bicinchoninic acid (BCA) protein assay (Pierce, Rockford, IL, USA). For each sample, 5 μg of peptides per condition (or pool) was desalted using C_18_ resin pipet tips (Zip Tip, Millipore Corp, Billerica, MA, USA) according to the manufacturer’s instructions. The eluted fractions were pooled and dried using a speed-vacuum at room temperature. The dry pellets were stored at −20 °C until further analysis. Prior to injection onto the 2D*nano*UPLC system and MS^E^ analysis, 2.5 µg of the digested proteins were resuspended in 9 μL of 100 mM ammonium formate solution adjusted to pH 10. The samples were spiked with the MassPREP*™* Digestion Standard Mixture (MPDS mix) (Waters Corp., Milford, USA) which contains a mixture of yeast enolase (ENO1, P00924), rabbit glycogen phosphorylase b (GPB, P00489), yeast alcohol dehydrogenase (ADH, P00330) and bovine serum albumin (BSA, P02769) digest. The corresponding ADH quantity spiked per sample injection was 150 fmol.

#### LC–MS/MS analysis

All samples were injected on a 2D-*nano*Aquity UPLC (Waters, Corp., Milford, USA) coupled online with a *Q*-Tof Synapt HDMS™ G2 system (Waters, Corp, Milford, USA) using ion mobility as an additional separation. The chromatographic peptide separation was performed on the *nano*UPLC system using a similar 2D-run in a reversed phase pH 10/reversed phase pH 3 dilution configuration. Briefly, the samples were loaded at 2 μL/min (20 mM ammonium formate solution, pH 10) on the first column (X-Bridge BEH C18, 5 µm) and subsequently eluted in five steps (10, 14, 16, 20 and 65% acetonitrile). These five fractions were desalted on the trap column (Symmetry C18, 5, 180 µm, 20 mm) after a 10 times online dilution to pH 3 and subsequently separated on the BEH C18 1.7 µm analytical column (75 µm, 250 mm, Waters, Corp., Milford, USA). The trap column conditions were a 250 nL/min flow rate 250 nL/min a gradient from 97% solvent A to 65% solvent A in 90 min with solvent A: 0.1% (v/v) formic acid in water and solvent B: 0.1% (v/v) formic acid in acetonitrile. The mass spectrometer was operated in positive ion mode. The data acquisition was performed in the 50–1500 *m*/*z* range with a scan time of 0.6 s and collision energy voltages set in independent alternative scanning (MS^E^) mode. The IMS parameters used were an IMS cell pressure of 2.5 mbar, a variable IMS wave velocity ranging from 850 to 1200 m/s and a wave height of 40 V. The singly charged peak of polysiloxan at *m*/*z* 445.12003 was used as lock mass and spectra were calibrated manually post acquisition.

#### Data analysis

##### Raw data analysis, protein identifications and differential analysis

Raw data were processed (deconvoluted, deisotoped), proteins identified/quantified and the differential analysis using relative quantification were all performed with Nonlinear Dynamics’ Progenesis program, version 1.1.4.8.32.42.175 (Waters, Newcastle on Tyne, UK). The parameters used for data processing were as follows: MS–TOF resolution and chromatographic peak width were set to automatic, low-/elevated-energy detection thresholds was 250/100 counts respectively and identification intensity threshold was set to 1500. For protein identifications, the UniProt human database without isoform was used as the reference (canonical sequence data with 20,280 entries, UniProt release 2011_12—December 14, 2011). The search parameters used were as follows: carbamidomethylation (C) as fixed modification, oxidation (M) and phosphorylation (STY) of peptides as variable ones, maximum two possible trypsin miss-cleavages was allowed, minimum fragment ion matches per peptide was set at 3, the minimum fragment ion matches per protein was set at 7 with minimum two peptides matches per protein (irrespective of possible peptide sequence redundancy). The maximum false positive discovery rate (FDR) on protein identification was set to 4%.

Differential analysis on nonlinear dynamics’ progenesis was done not considering protein isoforms and these were “grouped” in only one protein identification hit. Only the proteins identified in minimum 80% of samples whatever disease group was considered for further analysis. A normalisation was applied using the peptides identified as unique to one protein (=non-conflicting features). The Anova test was applied to compare the four categories of samples (DH vs. DI vs. pT1N0M0 vs. pT2N0M0) as well as the two cancer stages (pT1N0M0 vs. pT2N0M0). The proteins identified with at least two peptides, showing significant p value (≤0.05) and an absolute fold change (Fc) ≥2 were selected and considered as potential biomarkers.

### Selection of the three proteins of interest

The selection of the three proteins of interest was done according to the experimental results obtained (high Fc and significance in the two comparisons addressed: pT1N0M0 vs. pT2N0M0 and DH vs. DI vs. pT1N0M0 vs. pT2N0M0) and according to data available in the literature.

### IHC validation of three proteins of interest

#### Immunohistochemistry of kininogen-1, transport protein Sec24C and olfactomedin-4

**Fig. 2:**
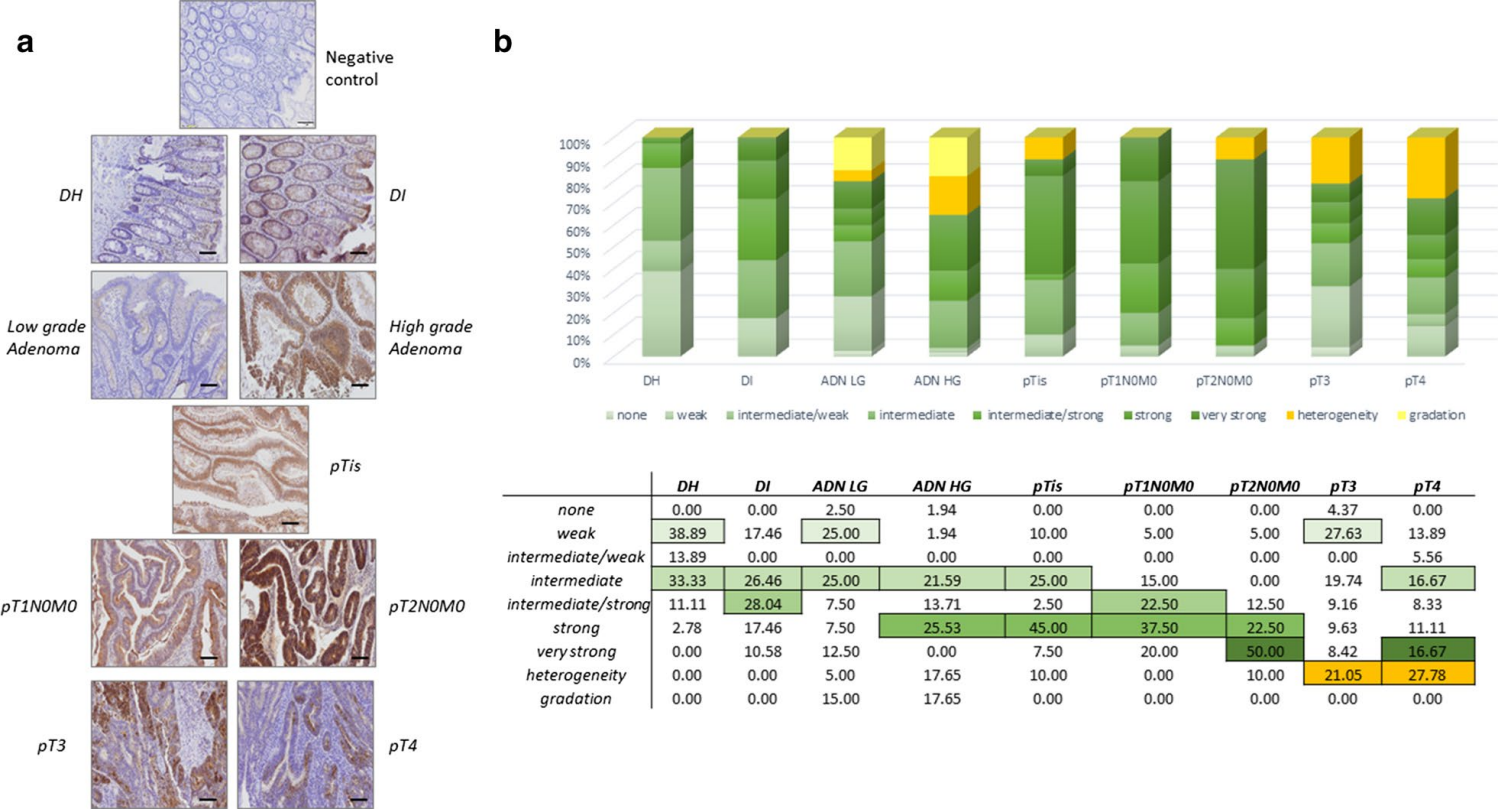
Immunohistochemical staining results obtained for OLFM4. **a** Representative staining pictures for OLFM4 in diverticular disease tissues (DI and DH), adenoma low grade (ADN LG)/high grade (ADN HG) and adenocarcinoma (ADK) tissues (*scale bar* 100 µm). **b** Quantification summary for OLMF4. The relative percentage calculated over all the categories of signal (from “none” to “gradation”) are detailed in the table. The highest value obtained is *underlined* in *colour* in each patient group represented by histograms and significant differences were obtained between DH versus pTis (p < 0.05), DH versus pT1 (p < 0.001), DH versus pT2 (p < 0.001), ADN low grade versus pT2 (p < 0.01), DI versus pT2 (p < 0.05) and pT2 versus pT3 (p < 0.01). *DH* diverticulitis (adjacent normal tissue), *DI* diverticulitis inflammatory (diverticulitis zone itself), *ADK* adenocarcinome, *ADN LG* adenoma low grade, *ADN HG* adenoma high grade

**Fig. 3:**
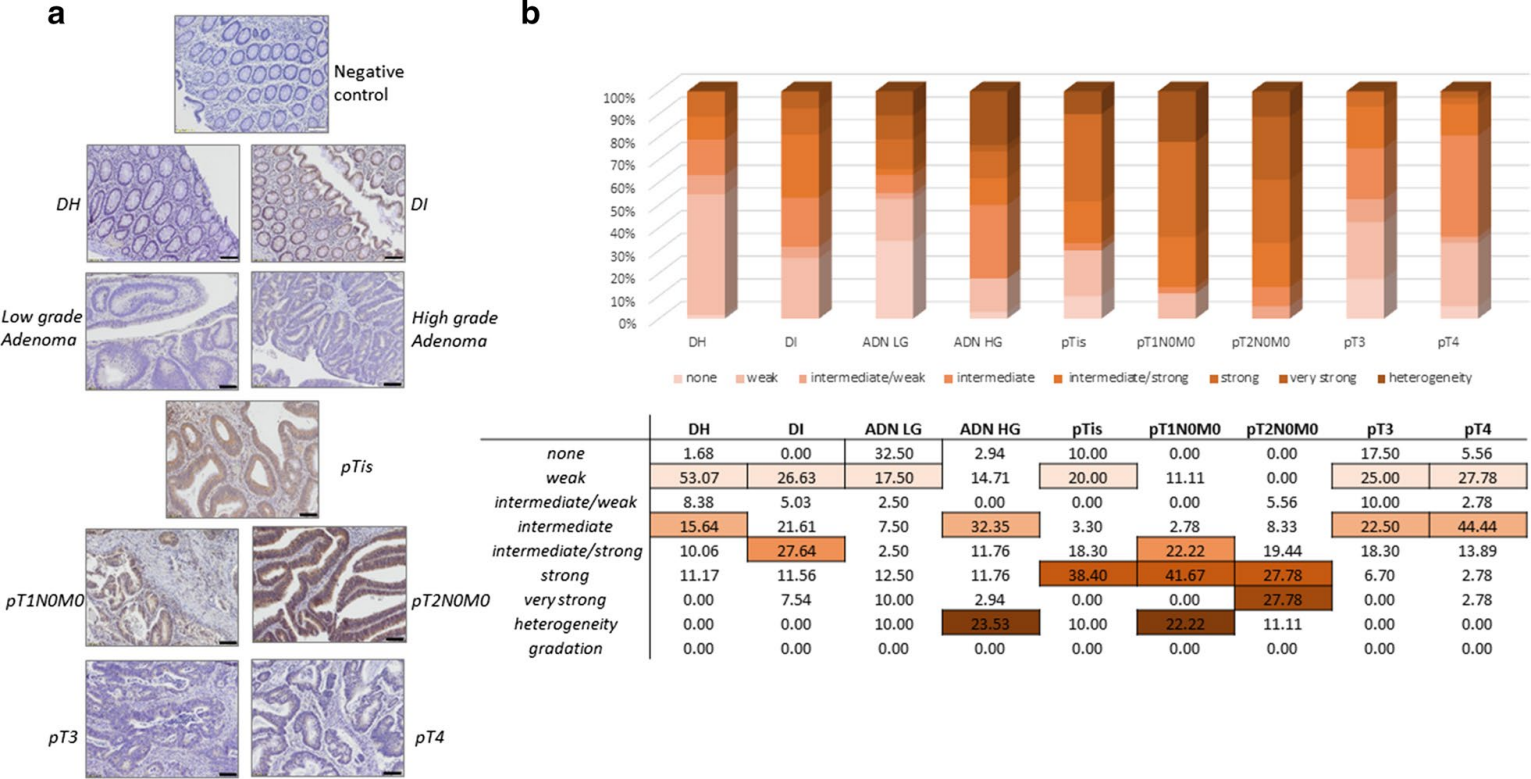
Immunohistochemical staining results obtained for Sec24C. **a** Representative staining pictures for Sec24C in diverticular disease tissues (DI and DH), adenoma low grade (ADN LG)/high grade (ADN HG) and adenocarcinoma (ADK) tissues (*scale bar* 100 µm). **b** Quantification summary for Sec24C. The relative percentage calculated over all the categories of signal (from “none” to “gradation”) are detailed in the table. The highest value obtained is *underlined* in *colour* in each patient group represented by histograms and significant differences were obtained between DH versus pT1N0M0, DH versus pT2N0M0, ADN low grade versus pT1 (p < 0.05), ADN low grade versus pT2 (p < 0.001), pT1 versus pT3 (p < 0.01), pT1 versus pT4 (p < 0.05), pT2 versus pT3 (p < 0.001), pT2 versus pT3 (p < 0.001), DI versus pT2 (p < 0.05) *DH* diverticulitis (adjacent normal tissue), *DI* diverticulitis inflammatory (diverticulitis zone itself), *ADK* adenocarcinome, *ADN LG* adenoma low grade, *ADN HG* adenoma high grade

**Fig. 4:**
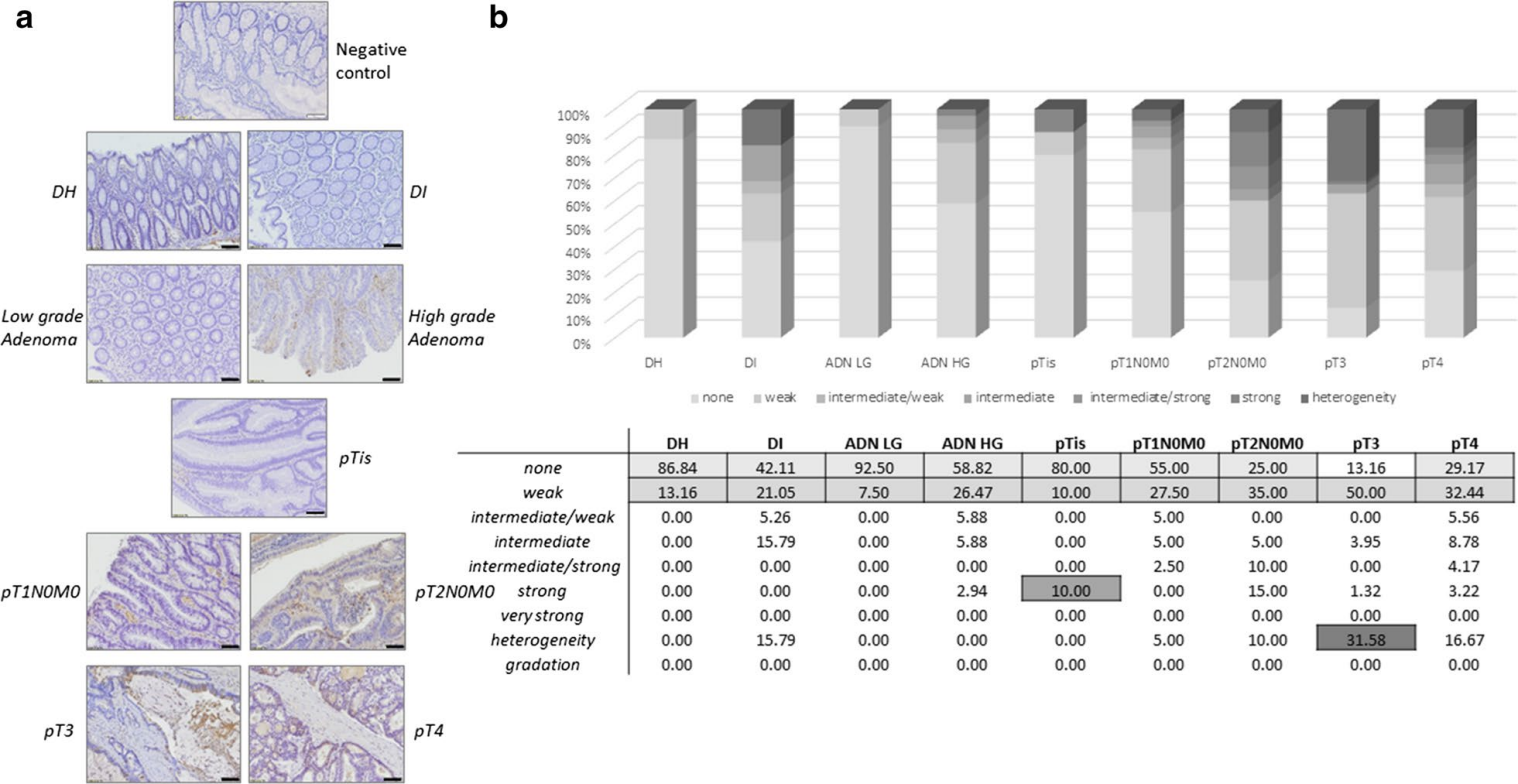
Immunohistochemical staining results obtained for KNG1. **a** Representative staining pictures for KNG1 in diverticular disease tissues (DI and DH), adenoma low grade (ADN LG)/high grade (ADN HG) and adenocarcinoma (ADK) tissues (*scale bar* 100 µm). **b** Quantification summary for KNG1. The relative percentage calculated over all the categories of signal (from “none” to “gradation”) are detailed in the table. The highest value obtained is *underlined* in *colour* in each patient group represented by histograms and significant differences were obtained between DH versus pT2 (p < 0.001), DH versus pT3 (p < 0.05), DH versus pT4 (p < 0.01), ADN low grade versus pT2 (p < 0.001), ADN low grade versus pT3 (p < 0.05), ADN low grade versus pT4 (p < 0.01), ADN high grade versus pT2 (p < 0.05), pT1 versus pT2 (p < 0.05) DI versus ADN low grade (p < 0.05). *DH* diverticulitis (adjacent normal tissue), *DI* diverticulitis inflammatory (diverticulitis zone itself), *ADK* adenocarcinome, *ADN LG* adenoma low grade, *ADN HG* adenoma high grade

The 6 µm section slices mounted on glass slide were heated at 60 °C, deparaffinized in xylene and rehydrated in graded isopropanol baths. Antigen retrieval was performed with a steamer for 10 min in target retrieval solution (DAKO) for all three antibodies. Endogenous peroxidase activity was quenched by incubation with 3% hydrogen peroxide for 10 min, followed by incubation with Protein block serum-free ready-to-use (DAKO) for 30 min to block nonspecific binding. The sections were subsequently incubated with the primary antibodies in appropriate dilutions overnight at 4 °C for the anti-olfactomedin-4 antibody, cat ab85046 (ABCAM) and anti-Sec24C antibody, cat ab122635 (ABCAM). The incubation for kininogen-1 (KNG1) IHC was performed 1 h at room temperature using anti-kininogen antibody, cat sc-25799 (Santa-Cruz Biotechnology). The sections were incubated for 30 min with EnVision + System-HRP labelled polymer anti-rabbit (DAKO). The chromogen used was 3,3′-diaminobenzidine and counterstaining was done with hematoxylin. The isotype controls used for all three antibodies were performed with the same method as used for each specific IHC, see in Figs. 2, 3 and 4 the illustrating picture (named negative control).

##### Quantitative analysis of IHC staining and statistics

IHC results were evaluated in at least three areas by two independent scorers (by FQC, CM) not trained in anatomopathology and without prior knowledge of clinical data and proteomic results. Immuno-stained sections were scored positive if epithelial cells showed specific staining in the cytoplasm, in the plasma membrane and/or in the nucleus. A semi-quantitative score was determined by estimating the percentage of the stained cells and the averaged signal intensity. The scale used was ranging from none (score 0), weak (score 1), intermediate/weak (score 2), intermediate (score 3), intermediate/strong (score 4), strong (score 5) to very strong (score 6). Two particular patterns of staining were also identified: one was termed “gradation” and refers to an increasing gradient of intensity progressing from the base of the crypt to the upper epithelium border. The second pattern was named “heterogeneity” and was used to describe areas where some cells were positive and some were negative. The semi-quantitative scores attributed to these particular patterns were based on the average value of the scores corresponding to the different fields analysed.

Statistical analyses were achieved using GraphPad Instat vs 3 and Prism softwares vs 6. The Kruskal–Wallis test was used to compare each groups scores as defined above ranging from 0 to 6. Results were considered significant if the associated p value was <0.05.

### Gene expression meta-analysis using published microarray data

The meta-analysis of gene expression obtained from public microarray datasets was done using the gene expression commons (GEXC) platform [12]. The independent datasets of adequate clinical scopes included were GSE13471 [13], GSE13428 [14] and GSE62932, including the tissues analysis of 96 patients. This platform aims at normalising microarray data against the common reference to obtain an absolute gene expression level within a specific tissue or organism. All gene expression datasets included were acquired using AffymetrixU133Plus2.0 human microarray and were obtained from NIH gene expression omnibus (accession no. GSE). We included only datasets originated from CRC or normal tissues (using Caucasian patients with known disease classification or classifications similar to our study). The CRC staging classification used for GSE62932 was distinct from the one used in our study using grades instead of pTNM stages. The data were normalised and the “standard robust average algorithm” generated the reference absolute “set expression level” [15]. The StepMiner algorithm allowed to assign to each protein a specific threshold which was used for result interpretation [16]. We built several populations and several models for the selected protein/gene candidates.

### GO analysis

The GO analyses were performed using STRING db (v. 10.0) and PANTHER db (V.11.0). Only the proteins found significant in “DH versus DI versus pT1N0M0 versus pT2N0M0” and “pT1N0M0 versus pT2N0M0” and showing a minimum Fc of 2 were considered for GO analyses.

## Results

### Proteomic discovery on early CRC stages

#### Raw data and technical results

In order to detect proteins differentially distributed through early CRC stages and controls, we generated protein extracts from the 76 FFPE tissues regrouped into the four categories compared, as illustrated Fig. 1. Each pool was analysed as a biological replicate and the raw data file generated have been deposited to the ProteomeXchange Consortium (http://proteomecentral.proteomexchange.org) via the PRIDE partner repository [17] with the dataset identifier PXD005735 (Additional file 1: Table S1).

The percentage of variability obtained on the three proteins of the MPDS mix spiked and established using normalisation on ADH, was lower than 30% which is in agreement with the expected maximum variability of the technique [18].

#### Protein identifications/quantitations and differential analysis

We obtained 5258 proteins identified through 80% of the samples analysed and 3547 proteins quantified in the four groups (DH, DI, pT1N0M0 and pT2N0M0). We performed several comparisons: DH versus DI versus pT1N0M0 versus pT2N0M0 and pT1N0M0 versus pT2N0M0. A total of 561 proteins were found significant in the four group comparison. We selected the 120 proteins that were significant in both comparisons, see Additional file 2: Table S2. The proteins significant in pT1N0M0 versus pT2N0M0 are likely associated to CRC progression. Ten percent of these proteins have been previously associated with cancer development or CRC [19–21] and are in bold in Additional file 2: Table S2. This also includes the results of two proteomic studies performed by two other groups [22, 23]. The proteins significantly differentially distributed between normal enterocytes and cancer cells isolated by laser microdissection are in blue [22] and the proteins significantly different between stromal cells of colon adenocarcinoma and non-neoplastic colon mucosa are in green [23] in Additional file 2: Table S2.

### Selection of some potential protein biomarkers and their validation

To partially validate our proteomic results, we selected three proteins (in red in Additional file 2: Table S2) that were found differentially distributed in one or both comparisons: (pT1N0M0 vs. pT2N0M0) and (DH vs. DI vs. pT1N0M0 vs. pT2N0M0). The olfactomedin-4 (OLFM4) showed a maximum Fc of 28.18 (p value = 0.039), protein transport protein Sec24C (Sec24C) showed a maximum Fc of 33.14 (p value = 0.028) in the four group comparison. The KNG1 present a maximum Fc of 3.92 (p value = 0.037) in the pT1N0M0 versus pT2N0M0 analysis. The selection of these three proteins for IHC validation was done according to the value of the Fc and p value obtained, on the availability of commercial IHC antibodies as well as data available in the literature. Indeed, some of these were not yet or seldom reported in CRC despite often associated to tumorigenesis or cancer progression in general and were more abundant in tumor than in normal tissues.

#### Tissue distribution of OLFM4

Representative pictures of OLFM4 staining are shown in Fig. 2a for each pathology group. The results of the semi-quantitative analysis of OLFM4 staining are summarised in Fig. 2b. The chart computes the relative percentages obtained in the different categories defined for the different disease groups. The statistical analysis done using the semi-quantitative scores (ranging from 0 to 6) showed significant differences.

OLFM4 staining was present in the basal crypt cells and in luminal surface epithelium. It was detected in the stromal cells such as inflammatory cells and fibroblasts, with percentages and intensities both increasing from precancerous to cancerous tissues. This increase was only observed up to CRC stage 2 and was less intense in CRC stages 3 and 4. Some epithelial cells in colonic mucosa distant from the original tumor showed strong positive signal. OLFM4 staining in epithelial or stromal cells was not significantly correlated with age, gender, N or M classification or tumor localisation.

#### Tissue distribution of Sec24C

Representative pictures of Sec24C staining are shown in Fig. 3a for each pathology group. The results of Sec24C semi-quantitative analysis are summarised in Fig. 3b. The chart computes the relative percentages obtained in the different categories defined for the different groups. Sec24C was found significantly more abundant in epithelial cells of pT1 and pT2N0M0 compared to DH (p value <0.01 and p value <0.001, respectively). Sec24C was also more abundant in the cells of the crypts and at the surface epithelium than in the chorion. The statistical analysis done using semi-quantitative scores provided significant differences.

A more pronounced signal (percentage of cells or labelling intensity within positive cells) for Sec24C was observed in the stroma of diverticular disease tissues (in both DH and DI) and in early CRC stages (pT1–pT2). The inflammatory cells within the stroma that were positive were predominantly macrophages with a positive staining of the cytoplasmic vesicles. Moreover the neoplastic cells progressing as a migrating front also showed an important positive Sec24C expression.

#### Tissue distribution of KNG1

Representative pictures of KNG1 staining are shown in Fig. 4 for each pathology group. The results of the KNG1 semi-quantitative analysis are summarised in the Fig. 4b. The chart computes the relative percentages obtained in the different categories defined for the different groups. The KNG1 expression was significantly increased in cancerous lesions compared to normal and dysplastic tissues.

### GEXC meta-analysis

**Fig. 5:**
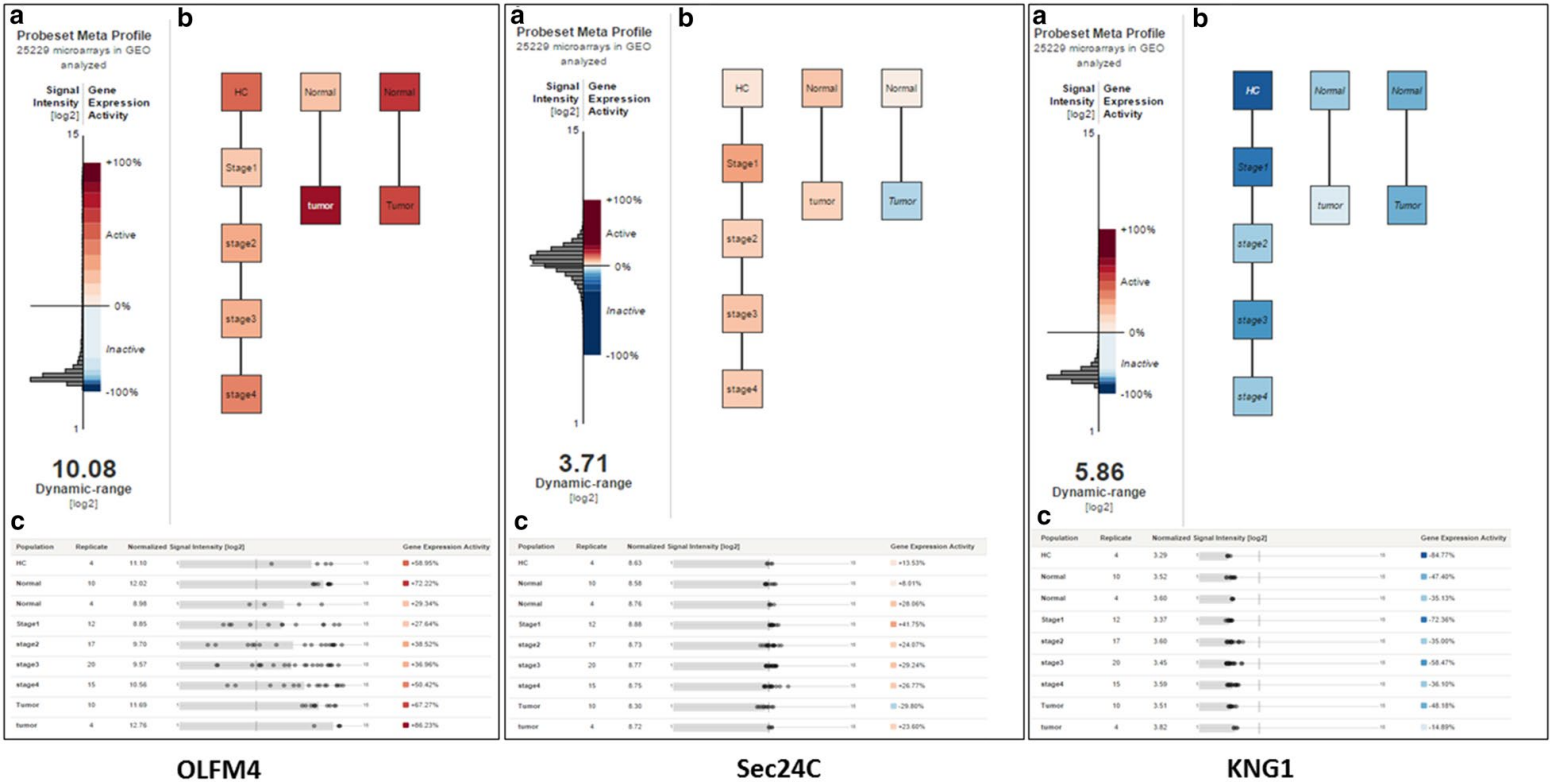
GEXC meta-analysis of OLFM4, Sec24C and KNG1. **a** Graphical representation of the Probeset meta profile obtained for each transcript. The Y axis represents the expression level (normalised in log2 scale) with an *histogram graph* illustrating the distribution of the gene expression within the *colour* gradient spread over the whole distribution of the transcript calculated with the patient data set included. The *colour bar* gradient displays the gene expression activity and the distribution of percentiles (down regulated in *blue*, upregulated in *red*). **b** Absolute gene expression of OLFM4, Sec24C and KNG1: each model is figured by *boxes* and *connecting lines* representing the biological context and relationships among populations of patients. A population contains several microarray data. “Tumor” contains colorectal adenocarcinoma data (stage non specified), “normal” contains the matched normal colon data, “HC” refers to healthy control data (obtained using colon tissue), stage 1–4 refer to the CRC dataset with the corresponding stages. **c** Number of replicates included into the considered populations and the distributions of the specific protein expression levels within the evaluated models. *HC* healthy control, *GEO* gene expression omnibus

The results obtained for the transcriptional expression level of the proteins discriminant in the comparison pT1N0M0 versus pT2N0M0 are summarised in Additional file 2: Table S2. The GEXC analysis generated transcriptional expression results for OLFM4, Sec24C and KNG1 that are illustrated Fig. 5a–c respectively. The expression of genes do not always correlate perfectly with the protein levels observed, which can be also explained by the difference of CRC stages—grades studied in the series of patients included in these analyses. However Sec24C and KNG1 gene expression levels increased along with the corresponding protein levels in healthy tissues and in CRC stages 1 and 2. The Sec24C decrease obtained in later CRC stages was in accordance with what was observed in our IHC results. Hence similar distribution tendencies in the two independent cohorts of tissue samples could be obtained. However, an inverse tendency was obtained for OLFM4 gene expression and protein abundance. The expression levels of the three genes can be explore at: https://gexc.stanford.edu/models/1444/genes/OLFM4?q=OLFM4, https://gexc.stanford.edu/models/1444/genes/SEC24C?q=sec24c, https://gexc.stanford.edu/models/1444/genes/KNG1.

## Discussion

The understanding of the mechanisms and the identification of markers of CRC development and progression are both very important; especially at early stages when patients are still asymptomatic. Hence we focused on the comparison of tissue proteomes of two early CRC stages (pT1N0M0 and pT2N0M0) with those of normal and inflamed control tissues (DI and DH). Inflamed tissues inclusion in the experimental design allowed us to identify potential inflammation-related proteins among the potential early CRC markers highlighted. Our proteomic study showed altered protein expression in early CRC tissues, a focus rarely addressed by proteomics. We showed by both proteomics and IHC the altered tissue expression of OLFM4 and KNG1, especially in early CRC stages. The abnormal production of Sec24C in these early CRC stages was also reported for the first time.

We previously demonstrated the ability to obtain an efficient and repeatable protein extraction using limited quantities of FFPE material before data-independent label-free analysis [24]. We performed macrodissection to favor the selection of cells of interest, removing abundant stromal tissue surrounding the tumor and decreasing the impact of response factors originated from neighboring tissues. Moreover the proteomic technology used was robust and suited to address such clinical question thanks to the high resolution, high speed of the mass spectrometers used in combination with the advanced computational methods allowing confident protein identifications and differential analysis [25–28]. We used two dimensional nanoUPLC enabling efficient chromatographic peptides separation while keeping data acquisition length reasonable. We could reach repeatable signal as technically expected using data independent analysis with MSE on Synapt G2, combining high resolution with ion mobility separation as supplementary dimension [18].

The 561 potentials biomarkers highlighted (Fc ≥2) in the four group comparison corresponds to 10.67% (15.81%) of the proteome identified (quantified). This percentage of potential candidates selected by label-free proteomics albeit applied on FFPE samples is in agreement with proteomic results of other teams using other MS instruments, statistics and other sample types and biological matrix [29, 30].

Based on proteomic results and data available in the literature, we selected three proteins found differentially abundant in early CRC tissues for IHC confirmation. We could characretise their IHC profiles in a second and larger independent set of samples/patients, including normal tissues, adenoma, pTis and the four progressive CRC stages (from pT1NM to pT4NM). Moreover, their respective transcriptional expression levels evaluated using GEXC on a third independent set of samples/patients also corroborated some of our proteomic and IHC findings.

OLFM4 is generally expressed in the basal crypt cells and is considered as a specific stem cell protein [31–35]. It is involved in cancer development via antiapoptotic action, in cell proliferation, cell adhesion and metastasis [36–38]. In our study, IHC OLFM4 level appeared significantly higher in the epithelial cells of pTis, pT1 and pT2 while it was significantly lower in pT3 (and even lower in pT4) as previously shown [39]. Interestingly, OLFM4 was also detected in stromal cells such as inflammatory cells and fibroblasts and this with a higher intensity in adenoma and adenocarcinoma tissues. But again with lower signals for pT3 and pT4 compared to other tissues. In the study of Seko et al. [38], 36% of CRC cases tested showed an OLFM4 tumor cytoplasmic staining. Moreover they observed extracellular staining far from the original tumor, however this could not be confirmed in this study. OLFM4 is secreted and may be detected in serum and plasma therefore representing a good candidate CRC marker as previously suggested for gastric cancer [35, 36].

Sec24C is an essential coat protein II (COPII) component and COPII is involved in protein transports from the endoplasmic reticulum (ER). Recently the AKT kinase was proposed as a new player in the control of ER protein transport [37]. Indeed AKT phosphorylates the two Sec24 isoforms (Sec24D and Sec24C) consequently affecting transport. AKT leads to cell survival, stimulates cell growth and increases proliferation. In CRC and other cancers, genetic aberrations lead to AKT hyperactivation, while adenoma tissues were found to overexpress AKT [40]. Finally inhibition of AKT decreases SEC24 protein levels [37]. The distribution of Sec24C that we observed showed a general increase in adenoma and early CRC stages, while its expression appeared decreased in more advanced CRC stages. This is in line with AKT overexpression known as an early event in colon carcinogenesis [40]. Altogether, these observations may provide further explanations of our results.

KNG1 is a cysteine proteinase inhibitor that can be cleaved into six subchains. It is implicated in blood coagulation and inflammatory response and was recently described as a serum biomarker for the early detection of advanced colorectal adenoma and CRC [41]. Indeed, KNG1 serum levels were lower in postoperative than in preoperative CRC patients. Their hypothesis to explain this observation was that an increased production of KNG1 derives from tumor tissues. Accumulating studies continue to demonstrate higher levels in different biological fluids (urine and sera) from different cancers [42, 43]. In this respect, our study confirmed that KNG1 was detected in FFPE tissue by proteomics and IHC with higher signal intensity and in more cells in the different CRC stages. Mostly in early CRC compared to adenoma and control tissues. Although its mechanistic role remains unclear in cancer, KNG1 could have antiangiogenic properties and inhibitory actions on proliferation of endothelial cells [44]. Previous studies have shown lower levels of KNG1 in urine or sera of ovarian carcinoma cases and cervical cancer patients respectively [45, 46]. But in these studies the protein detected was KNG1 light chain and not the entire protein. Hence detection of KNG1 as a whole or specifically its light chain could be futher investigated as promising targets for cancer diagnosis.

Interestingly OLFM4 and Sec24C showed a more abundant expression in early than in later CRC stages which is of particular value when aiming at early diagnosis. However their distributions at the systemic level should first be investigated to establish their value as potential early and non invasive CRC biomarkers.

This partial validation of our proteomic results as well as converging observations with other works in a related context [19–23] suggest that other proteins highlighted in this work might also be potential biomarkers associated to early CRC stages.

## Conclusion

Our data confirm the ability of a research strategy based on proteomic screening using FFPE tissue samples, followed by IHC confirmation on an independent set of samples/patients to identify potential protein markers associated to early CRC stages. We were able to confirm abnormal expression of OLFM4 and KNG1 in CRC tissues, especially in early pTNM CRC stages. This could also be observed but more moderately in precancerous lesions. We showed for the first time an overexpression of Sec24C in early stages and a decrease in later stages of CRC. Further studies on these proteins are warranted to better understand their role in CRC progression and their potential as early diagnostic tools.

## Additional files

**Additional file 1: Table S1.** The 5258 proteins found significant in the differential proteomic discovery study comparing DH *versus* DI *versus* pT1N0M0 *versus* pT2N0M0. Abbreviations: DH: diverticulitis (adjacent normal tissue), DI: diverticulitis inflammatory (diverticulitis zone itself), ADK: adenocarcinome.

**Additional file 2: Table S2.** The 121 proteins found as most discriminant in the DH *versus* DI *versus* pT1N0M0 *versus* pT2N0M0 comparison and common to the list of proteins found significant in the pT1N0M0 *versus* pT2N0M0 analysis are reported with p value, Fc and with the results of the analysis on GEXC. Abbreviations: DH: diverticulitis (adjacent normal tissue), DI: diverticulitis inflammatory (diverticulitis zone itself), ADK: adenocarcinome, GEXC: Gene Expression Commons, NR: Not relevant, Absent: Not available in the Data set, yes: the distribution of the gene expression between groups showed a similar tendency to the protein distribution obtained by proteomics, no: the distribution of the gene expression between groups did not showed a similar tendency to the protein distribution obtained by proteomics, The proteins previously associated with cancer development or CRC [19–22, 45] are in bold. Protein selected and validated in this paper are in red. Proteins found significant between normal enterocytes and cancer cells are in blue. Proteins found significant between stromal cells of colon adenocarcinoma and non-neoplastic colon mucosa are in green.

## Abbreviations

ADN: adenoma
ADN LG: adenoma low grade
ADN HG: adenoma high grade
ADK: adenocarcinoma
BCA: bicinchoninic acid
COPII: coat protein II component
CRC: colorectal cancer
DH: diverticulitis healthy
DI: diverticulitis inflammatory
ER: endoplasmic reticulum
FASP: filter aided sample preparation
Fc: fold change
FFPE: formalin fixed paraffin embedded
FOBT: faecal occult blood test
FIT: faecal immunochemical test
GEO: gene expression omnibus
GEXC: gene expression commons
HC: healthy controls
H&E: hematoxylin and eosin
KNG1: kininogen-1
OLFM4: olfactomedin-4
SEC24C: protein transport protein Sec24C

## References

[1] Peters U, Bien S, Zubair N (2015). Genetic architecture of colorectal cancer. Gut.

[2] Allen JI (2015). Quality measures for colonoscopy: where should we be in 2015?. Curr Gastroenterol Rep.

[3] Hassan C, Giorgi Rossi P, Camilloni L, Rex DK, Jimenez-Cendales B, Ferroni E, Borgia P, Zullo A, Guasticchi G (2012). Meta-analysis: adherence to colorectal cancer screening and the detection rate for advanced neoplasia, according to the type of screening test. Aliment Pharmacol Ther.

[4] Langan RC, Mullinax JE, Raiji MT, Upham T, Summers T, Stojadinovic A, Avital I (2013). Colorectal cancer biomarkers and the potential role of cancer stem cells. J Cancer.

[5] Labianca R, Nordlinger B, Beretta GD, Brouquet A, Cervantes A, ESMO Guidelines Working Group (2010). Primary colon cancer: ESMO Clinical Practice Guidelines for diagnosis, adjuvant treatment and follow-up. Ann Oncol.

[6] Balmana J, Castells A, Cervantes A (2010). Familial colorectal cancer risk: ESMO clinical practice guidelines. Ann Oncol.

[7] Glimelius B, Pahlman L, Cervantes A (2010). Rectal cancer: ESMO clinical practice guidelines for diagnosis, treatment and follow-up. Ann Oncol.

[8] Schlemper RJ, Riddell RH, Kato Y, Borchard F, Cooper HS, Dawsey SM, Dixon MF, Fenoglio-Preiser CM, Flejou JF, Geboes K (2000). The Vienna classification of gastrointestinal epithelial neoplasia. Gut.

[9] Bancroft JD, Gamble M (2007). Theory and Practice of Histological Techniques.

[10] Marée R, Stévens B, Rollus L, Rocks N, Lopez XM, Salmon I, Cataldo D, Wehenkel L (2013). A rich internet application for remote visualization and collaborative annotation of digital slides in histology and cytology. Diagn Pathol.

[11] Quesada-Calvo F, Bertrand V, Longuespée R, Delga A, Mazzucchelli G, Smargiasso N, Baiwir D, Delvenne P, Malaise M, De Pauw-Gillet M-C (2015). Comparison of two FFPE preparation methods using label-free shotgun proteomics: application to tissues of diverticulitis patients. J Proteomics.

[12] Seita J, Sahoo D, Rossi DJ, Bhattacharya D, Serwold T, Inlay MA, Ehrlich LI, Fathman JW, Dill DL, Weissman IL (2012). Gene expression commons: an open platform for absolute gene expression profiling. PLoS ONE.

[13] Irizarry RA, Ladd-Acosta C, Wen B, Wu Z, Montano C, Onyango P, Cui H, Gabo K, Rongione M, Webster M (2009). The human colon cancer methylome shows similar hypo- and hyper-methylation at conserved tissue-specific CpG island shores. Nat Genet.

[14] Lin G, He X, Ji H, Shi L, Davis RW, Zhong S (2006). Reproducibility probability score—incorporating measurement variability across laboratories for gene selection. Nat Biotechnol.

[15] Bolstad BM, Irizarry RA, Astrand M, Speed TP (2003). A comparison of normalization methods for high density oligonucleotide array data based on variance and bias. Bioinformatics.

[16] Sahoo D, Dill DL, Tibshirani R, Plevritis SK (2007). Extracting binary signals from microarray time-course data. Nucleic Acids Res.

[17] Vizcaino JA, Cote RG, Csordas A, Dianes JA, Fabregat A, Foster JM, Griss J, Alpi E, Birim M, Contell J (2013). The PRoteomics IDEntifications (PRIDE) database and associated tools: status in 2013. Nucleic Acids Res.

[18] Vissers JP, Langridge JI, Aerts JM (2007). Analysis and quantification of diagnostic serum markers and protein signatures for Gaucher disease. Mol Cell Proteomics.

[19] Roblick UJ, Hirschberg D, Habermann JK, Palmberg C, Becker S, Kruger S, Gustafsson M, Bruch HP, Franzen B, Ried T (2004). Sequential proteome alterations during genesis and progression of colon cancer. Cell Mol Life Sci.

[20] Jimenez CR, Knol JC, Meijer GA, Fijneman RJ (2010). Proteomics of colorectal cancer: overview of discovery studies and identification of commonly identified cancer-associated proteins and candidate CRC serum markers. J Proteomics.

[21] Albrethsen J, Knol JC, Piersma SR, Pham TV, de Wit M, Mongera S, Carvalho B, Verheul HM, Fijneman RJ, Meijer GA, Jimenez CR (2010). Subnuclear proteomics in colorectal cancer: identification of proteins enriched in the nuclear matrix fraction and regulation in adenoma to carcinoma progression. Mol Cell Proteomics.

[22] Wisniewski JR, Dus-Szachniewicz K, Ostasiewicz P, Ziolkowski P, Rakus D, Mann M (2015). Absolute proteome analysis of colorectal mucosa, adenoma, and cancer reveals drastic changes in fatty acid metabolism and plasma membrane transporters. J Proteome Res.

[23] Mu Y, Chen Y, Zhang G, Zhan X, Li Y, Liu T, Li G, Li M, Xiao Z, Gong X, Chen Z (2013). Identification of stromal differentially expressed proteins in the colon carcinoma by quantitative proteomics. Electrophoresis.

[24] Quesada-Calvo F, Bertrand V, Longuespee R, Delga A, Mazzucchelli G, Smargiasso N, Baiwir D, Delvenne P, Malaise M, De Pauw-Gillet MC (2014). Comparison of two FFPE preparation methods using label-free shotgun proteomics: application to tissues of diverticulitis patients. J Proteomics.

[25] Trudgian DC, Ridlova G, Fischer R, Mackeen MM, Ternette N, Acuto O, Kessler BM, Thomas B (2011). Comparative evaluation of label-free SINQ normalized spectral index quantitation in the central proteomics facilities pipeline. Proteomics.

[26] Mann M, Kulak NA, Nagaraj N, Cox J (2013). The coming age of complete, accurate, and ubiquitous proteomes. Mol Cell.

[27] Richardson K, Denny R, Hughes C, Skilling J, Sikora J, Dadlez M, Manteca A, Jung HR, Jensen ON, Redeker V (2012). A probabilistic framework for peptide and protein quantification from data-dependent and data-independent LC–MS proteomics experiments. OMICS.

[28] Geromanos SJ, Vissers JP, Silva JC, Dorschel CA, Li GZ, Gorenstein MV, Bateman RH, Langridge JI (2009). The detection, correlation, and comparison of peptide precursor and product ions from data independent LC–MS with data dependant LC–MS/MS. Proteomics.

[29] Luebker SA, Wojtkiewicz M, Koepsell SA (2015). Two methods for proteomic analysis of formalin-fixed, paraffin embedded tissue result in differential protein identification, data quality, and cost. Proteomics.

[30] Tanca A, Pagnozzi D, Burrai GP, Polinas M, Uzzau S, Antuofermo E, Addis MF (2012). Comparability of differential proteomics data generated from paired archival fresh-frozen and formalin-fixed samples by GeLC–MS/MS and spectral counting. J Proteomics.

[31] Jang BG, Kim HS, Kim KJ, Rhee YY, Kim WH, Kang GH (2015). Distribution of intestinal stem cell markers in colorectal precancerous lesions. Histopathology..

[32] van der Flier LG, Haegebarth A, Stange DE, van de Wetering M, Clevers H (2009). OLFM4 is a robust marker for stem cells in human intestine and marks a subset of colorectal cancer cells. Gastroenterology.

[33] Zhang J, Liu WL, Tang DC, Chen L, Wang M, Pack SD, Zhuang Z, Rodgers GP (2002). Identification and characterization of a novel member of olfactomedin-related protein family, hGC-1, expressed during myeloid lineage development. Gene.

[34] Liu BG, Cao YB, Cao YY, Zhang JD, An MM, Wang Y, Gao PH, Yan L, Xu Y, Jiang YY (2007). Altered protein profile of lymphocytes in an antigen-specific model of colitis: a comparative proteomic study. Inflamm Res.

[35] Oue N, Sentani K, Noguchi T, Ohara S, Sakamoto N, Hayashi T, Anami K, Motoshita J, Ito M, Tanaka S (2009). Serum olfactomedin 4 (GW112, hGC-1) in combination with Reg IV is a highly sensitive biomarker for gastric cancer patients. Int J Cancer.

[36] Clemmensen SN, Glenthoj AJ, Heeboll S, Nielsen HJ, Koch C, Borregaard N (2015). Plasma levels of OLFM4 in normals and patients with gastrointestinal cancer. J Cell Mol Med..

[37] Sharpe LJ, Luu W, Brown AJ (2011). Akt phosphorylates Sec24: new clues into the regulation of ER-to-Golgi trafficking. Traffic.

[38] Seko N, Oue N, Noguchi T, Sentani K, Sakamoto N, Hinoi T, Okajima M, Yasui W (2010). Olfactomedin 4 (GW112, hGC-1) is an independent prognostic marker for survival in patients with colorectal cancer. Exp Ther Med.

[39] Besson D, Pavageau AH, Valo I, Bourreau A, Belanger A, Eymerit-Morin C, Mouliere A, Chassevent A, Boisdron-Celle M, Morel A (2011). A quantitative proteomic approach of the different stages of colorectal cancer establishes OLFM4 as a new nonmetastatic tumor marker. Mol Cell Proteomics.

[40] Roy HK, Olusola BF, Clemens DL, Karolski WJ, Ratashak A, Lynch HT, Smyrk TC (2002). AKT proto-oncogene overexpression is an early event during sporadic colon carcinogenesis. Carcinogenesis.

[41] Wang J, Wang X, Lin S, Chen C, Wang C, Ma Q, Jiang B (2013). Identification of kininogen-1 as a serum biomarker for the early detection of advanced colorectal adenoma and colorectal cancer. PLoS ONE.

[42] Navaneethan U, Lourdusamy V, Gk Venkatesh P, Willard B, Sanaka MR, Parsi MA (2015). Bile proteomics for differentiation of malignant from benign biliary strictures: a pilot study. Gastroenterol Rep.

[43] Liu W, Liu B, Cai Q, Li J, Chen X, Zhu Z (2012). Proteomic identification of serum biomarkers for gastric cancer using multi-dimensional liquid chromatography and 2D differential gel electrophoresis. Clin Chim Acta.

[44] Abdullah-Soheimi SS, Lim BK, Hashim OH, Shuib AS (2010). Patients with ovarian carcinoma excrete different altered levels of urine CD59, kininogen-1 and fragments of inter-alpha-trypsin inhibitor heavy chain H4 and albumin. Proteome Sci.

[45] Mu AK, Lim BK, Hashim OH, Shuib AS (2013). Identification of O-glycosylated proteins that are aberrantly excreted in the urine of patients with early stage ovarian cancer. Int J Mol Sci.

[46] Abdul-Rahman PS, Lim BK, Hashim OH (2007). Expression of high-abundance proteins in sera of patients with endometrial and cervical cancers: analysis using 2-DE with silver staining and lectin detection methods. Electrophoresis.

